# Safety and Efficacy of T-DM1 Plus Neratinib in Patients With Metastatic HER2-Positive Breast Cancer: NSABP Foundation Trial FB-10

**DOI:** 10.1200/JCO.19.00858

**Published:** 2019-08-23

**Authors:** Jame Abraham, Albert J. Montero, Rachel C. Jankowitz, Mohamad Adham Salkeni, Jan H. Beumer, Brian F. Kiesel, Fanny Piette, Laura M. Adamson, Rebecca J. Nagy, Richard B. Lanman, Jeff Sperinde, Weidong Huang, Carmen J. Allegra, Ashok Srinivasan, Ying Wang, Katherine L. Pogue-Geile, Peter C. Lucas, Samuel A. Jacobs

**Affiliations:** ^1^ NSABP Foundation, Pittsburgh, PA; ^2^ Cleveland Clinic, Taussig Cancer Institute, Cleveland, OH; ^3^ University of Pittsburgh School of Medicine, Pittsburgh, PA; ^4^ UPMC Hillman Cancer Center, Pittsburgh, PA; ^5^ West Virginia University, Morgantown, WV; ^6^ International Drug Development Institute, Louvain-la-Neuve, Belgium; ^7^ Guardant Health, Redwood City, CA; ^8^ Monogram Biosciences, Laboratory Corporation of America Holdings, South San Francisco, CA; ^9^ University of Florida Health, Gainesville, FL

## Abstract

**PURPOSE:**

Patients with human epidermal growth factor receptor 2 (HER2)–positive metastatic breast cancer eventually develop resistance to dual-antibody therapy with trastuzumab plus pertuzumab. Mechanisms of resistance have not been well elucidated. We evaluated the safety, tolerability, and efficacy of ado-trastuzumab emtansine (T-DM1) plus neratinib in patients who progressed on trastuzumab plus pertuzumab.

**PATIENTS AND METHODS:**

In this 3 + 3 dose-escalation study, patients with metastatic breast cancer who progressed on trastuzumab, pertuzumab, and a taxane were treated with T-DM1 at 3.6 mg/kg intravenously every 3 weeks and dose-escalating neratinib at 120, 160, 200, or 240 mg/d orally.

**RESULTS:**

Twenty-seven patients were treated across four dose-levels of neratinib. Dose-limiting toxicity in cycle 1 was grade 3 diarrhea in six patients and grade 3 nausea in one; no patient experienced grade 4 diarrhea, and there were no grade 5 toxicities. Other grade 3 to 4 toxicities included nausea (11%), dehydration (11%), electrolyte abnormality (19%), thrombocytopenia (15%), elevated transaminase levels (7%), and fatigue (7%). Twelve (63%) of 19 evaluable patients had an objective response. Responses occurred at all neratinib doses. Plasma cell–free DNA at baseline showed *ERBB2* (HER2) amplification in 10 of 27 patients. Deep and more durable responses occurred in patients with cell-free DNA *ERBB2* amplification. Two complete responders had high expression of total HER2 and p95HER2 in baseline tissue.

**CONCLUSION:**

We report the recommended phase II dose of T-DM1 3.6 mg/kg and neratinib 160 mg/d for this combination. Possible resistance mechanisms to HER2 antibodies may be loss of the HER2 receptor and high expression of p95HER2. These data provide the basis for an ongoing phase II study to better define the activity of this regimen.

## INTRODUCTION

Human epidermal growth factor receptor 2 (HER2) is overexpressed in 20% of breast cancers. The blocking of HER2 activity with trastuzumab, which binds to the extracellular domain IV of the HER2 receptor, prevents dimerization and inhibits downstream signaling, which results in cell cycle arrest and apoptosis and leads to improved outcomes for patients with HER2-positive disease.^1^ However, in women with metastatic breast cancers (MBCs), resistance usually occurs. To overcome resistance, additional anti-HER2 therapies, monoclonal antibodies, antibody-drug conjugates, and oral tyrosine kinase inhibitors (TKIs) have been developed. Pertuzumab, another monoclonal antibody, binds to extracellular domain II of HER2, prevents heterodimerization with HER3 and other HER receptors, and acts in a complementary fashion with trastuzumab to provide a more complete signaling blockade. This activity has been confirmed in clinical trials,^2-4^ which led to US Food and Drug Administration (FDA) approval of pertuzumab in metastatic and neoadjuvant settings.

Ado-trastuzumab emtansine (T-DM1), developed to treat trastuzumab-resistant patients, is a conjugated antibody composed of the cytotoxic agent DM1, a maytansinoid derivative attached to trastuzumab through a stable thioether linker.^5^ This first-in-class antibody-drug conjugate received US FDA approval for second-line anti-HER2 therapy on the basis of findings from the Trastuzumab Emtansine Versus Capecitabine Plus Lapatinib in Patients With Previously Treated HER2-Positive Advanced Breast Cancer (EMILIA) trial.^6^ Patients treated with multiple lines of anti-HER2 therapies had lower objective responses (ORs) and shorter progression-free survival with T-DM1 monotherapy than those observed in EMILIA.^7^ Although no prospective data of T-DM1 activity exist in patients whose cancer progresses on trastuzumab plus pertuzumab, a retrospective analysis showed tumor response to T-DM1 of less than 20%.^8^

Several TKIs show activity after progression on trastuzumab. Neratinib, an oral, small-molecule TKI of HER family members, was approved for extended adjuvant treatment in early-stage HER2-overexpressing/amplified breast cancer after completion of adjuvant trastuzumab-based therapy.^9^ In an earlier phase II study, neratinib as monotherapy in HER2-positive MBC demonstrated an overall response rate of 24% in trastuzumab-refractory patients and 56% in trastuzumab-naïve patients.^10^

To be effective, trastuzumab, pertuzumab, and T-DM1 must bind to the extracellular domain of HER2. The conjugated antibody T-DM1 binds to the HER2 receptor to allow for intracellular drug delivery of the potent cytotoxic agent DM1. In contrast, neratinib binds irreversibly to the intracellular ATP pocket of the HER2 tyrosine kinase domain. A potential advantage of the TKI is that truncated HER2 (p95HER2) lacks trastuzumab and pertuzumab binding sites while retaining the kinase domain, which potently drives downstream signaling. Expression of p95HER2 occurs in up to 30% of HER2-positive MBCs.^11^ Thus, high-p95HER2 expression is expected to result in resistance to trastuzumab, pertuzumab, and T-DM1 but retain sensitivity to several TKIs.

Taken together, T-DM1 plus neratinib combines agents with different mechanisms of action and toxicity profiles. As monotherapy, both agents have been shown to overcome trastuzumab resistance. The purpose of this study was to determine the safety and preliminary efficacy of the combination in patients previously treated with trastuzumab plus pertuzumab and to explore potential predictors of sensitivity and mechanisms of resistance.

## PATIENTS AND METHODS

### Patients

Eligible patients included women 18 years of age or older with an Eastern Cooperative Oncology Group performance status of 0 to 1; HER2-positive breast cancer (determined by local testing using the ASCO/College of American Pathologists HER2 test guideline)^12^; hormone receptor positivity or negativity; measurable metastatic disease by Response Evaluation Criteria in Solid Tumors (RECIST) version 1.1; and progression on anti-HER–based therapy with trastuzumab plus pertuzumab given in the neoadjuvant/adjuvant or first-line setting, irrespective of time to progression. Adequate hematologic parameters were absolute neutrophil count of 1,000/mm^3^ or greater, platelet count of 100,000/mm^3^ or greater, hemoglobin of 9 g/dL or greater, adequate renal function (defined as serum creatinine of less than or equal to 1.5 × the upper limit of normal [ULN]), and adequate hepatic function (defined as total bilirubin of less than or equal to 1.5 × ULN, AST and ALT of less than or equal to 2.5 × ULN, or 5 × ULN if liver metastases). Left ventricular ejection fraction (LVEF) of 50% or greater assessed by either two-dimensional echocardiogram or multiple-gated acquisition scan was required. Patients were excluded if they had received prior T-DM1 or TKI therapy; could not swallow medications; had symptomatic brain metastases (patients with known, stable brain metastases were allowed; CNS imaging in asymptomatic patients was not an entry requirement); active hepatitis B or C with abnormal liver function tests; persistent grade 2 or higher diarrhea; or active cardiac disease, including angina pectoris.

FB-10 was approved by the institutional review boards at participating institutions; written informed consent was required. The study was conducted according to Good Clinical Practice guidelines and the Declaration of Helsinki.

### Study Design

This phase Ib, multicenter, open-label, dose-escalation study evaluated T-DM1 plus neratinib in women with HER2-positive MBC. On day 1, patients received 3.6 mg/kg T-DM1 intravenously on a 3-week cycle and began daily oral neratinib in one of four dose-escalation cohorts (120, 160, 200, or 240 mg). Up to two dose reductions after cycle 1 were allowed for both T-DM1 and neratinib.

All patients who during cycle 1 experienced a dose-limiting toxicity (DLT) or received 75% of neratinib were considered evaluable for a DLT. Patients who did not complete cycle 1 for reasons other than toxicity were replaced. Patients who completed one cycle of therapy but not evaluated by imaging were considered nonevaluable for efficacy. DLT was defined as one or more of the following: diarrhea (any grade) with fever or dehydration requiring intravenous fluids, grade 3 diarrhea lasting more than 2 days on optimal medical therapy, grade 4 diarrhea of any duration, grade 3/4 neutropenia with fever, grade 4 neutropenia lasting more than 7 days, grade 4 thrombocytopenia, grade 3/4 nonhematologic toxicity (excluding grade 3 rash or allergic reaction/hypersensitivity), or toxicity-related delay of more than 2 weeks to initiate cycle 2. Progressive disease (PD) or grade 2 or higher toxicity, as defined for specific adverse events (AEs), would lead to permanent discontinuation of both T-DM1 and neratinib. The primary aim was to evaluate the safety of T-DM1 plus neratinib and to determine the recommended phase II dose (RP2D), which is the highest dose of neratinib with no more than one in six patients experiencing a DLT. Secondary end points were determination of rates of complete response (CR), partial response (PR), stable disease (SD), duration of response, overall toxicity, and exploratory studies to better understand therapy sensitivity or resistance. Because diarrhea is expected with neratinib, primary prophylaxis with loperamide was mandated to begin with the first dose and to continue throughout cycle 1 with subsequent titration of antidiarrheal therapy (guidance provided in the Data Supplement).

### Safety Assessment

Safety was assessed by physical examination, interim history, and laboratory assessment. Patients continued therapy until PD or discontinuation as a result of withdrawal, physician discretion, or toxicity. AE assessment occurred on days 1, 8, and 15 of cycle 1; on day 1 of each cycle; and for 30 days after therapy discontinuation or when alternate therapy began. AE reporting was according to the National Cancer Institute Common Terminology Criteria for Adverse Events (version 4.0). Patient safety and AEs were continuously monitored and reviewed by the NSABP medical review team and during weekly teleconferences with designated participating site staff and investigators.

### Response Assessment

Clinical activity assessed by tumor measurements using RECIST version 1.1 was performed at baseline and after every two cycles (6 weeks). Patients whose first scan (at 6 weeks) indicated CR, PR, or SD underwent a confirmatory scan at 12 weeks. After six cycles, responding patients were permitted to have the scanning interval increased to every third cycle.

### Correlative Studies

Blood samples for neratinib pharmacokinetic (PK) determination were required on day 1, cycle 1 (pretreatment), and day 1, cycle 2. Optional blood samples for more comprehensive neratinib PK determination were collected during cycle 1 at 1, 2, 4, 6, and approximately 22 to 24 hours after neratinib administration (n = 8). PK parameters were determined noncompartmentally with PK Solutions 2.0 (Summit Research Services, Montrose, CO). Plasma concentrations of neratinib were quantitated with an assay fully validated per US FDA guidance.^13^ Neratinib trough concentrations were extrapolated to the 24-hour value assuming a 14.9-hour half-life as reported previously.^14,15^ Statistical analyses were performed using SPSS version 22 software (IBM Corporation, Armonk, NY). Additional blood samples were collected in Streck tubes at baseline, during therapy, and at progression for cell-free DNA (cfDNA) and future analyses. cfDNA next-generation sequencing analyses were performed on all baseline samples (N = 27); selected day 1, cycle 2, samples (n = 9); and at progression (n = 2) at Guardant Health (Guardant360 assay), a Clinical Laboratory Improvement Amendments–certified, College of American Pathologists–accredited, New York State Department of Health–approved laboratory. The Guardant360 assay^16^ detects single-nucleotide variants, indels, fusions, and copy number alterations in 73 genes. For *ERBB2* amplification, a cutoff of more than 2.14 was used.

Two patients consented for optional tissue biopsies upon enrollment. These samples were assayed for total HER2 (H2T) using the HERmark assay^17^ and for p95HER2 using the VeraTag assay^18^ (Monogram Biosciences, South San Francisco, CA).

### Statistical Analyses

FB-10 was intended to determine the safety, tolerability, and RP2D of neratinib in combination with T-DM1. Categorical data were summarized by presenting frequencies and percentages. End point analyses were descriptive. No interim analyses were planned or conducted.

## RESULTS

### Patient Characteristics

From February 2015 to July 2017, five institutions enrolled 27 patients. Table 1 lists baseline patient characteristics. Median age was 48 years (range, 23 to 69 years); Eastern Cooperative Oncology Group performance status was 0 in 20 patients and 1 in seven. Hormone receptor status was positive in 15 patients and negative in 12. All patients had a history of HER2-positive disease and had received trastuzumab, pertuzumab, and taxane. Six patients with stable brain metastases were enrolled.

**TABLE 1.**
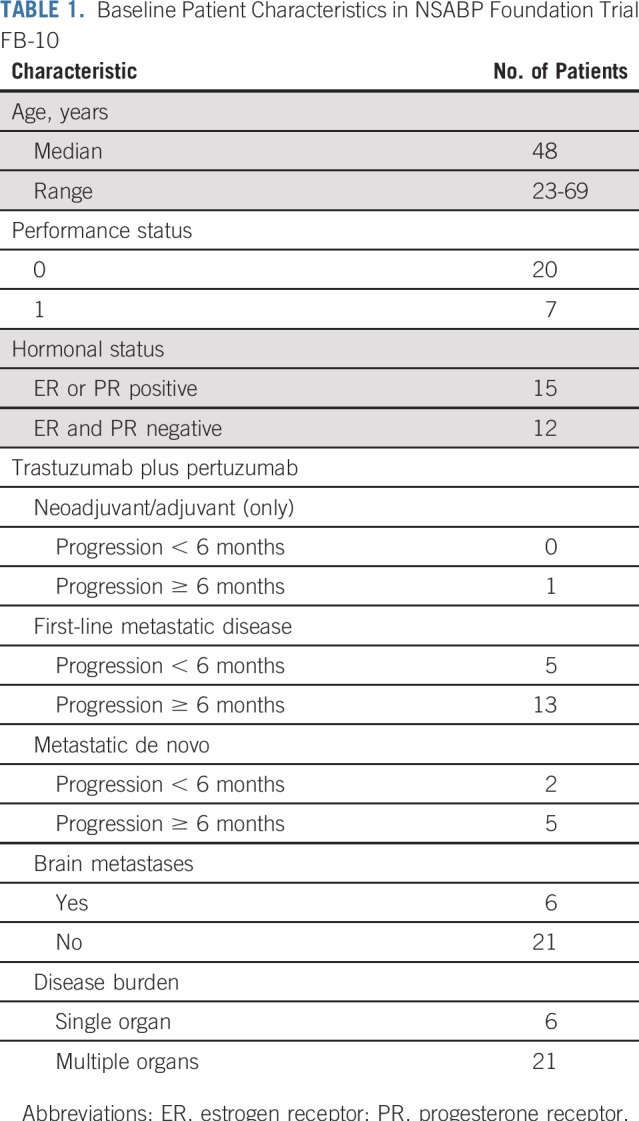
Baseline Patient Characteristics in NSABP Foundation Trial FB-10

### Safety Assessment

Twenty-five patients were evaluable for a DLT and 27 for AEs. All patients experienced at least one AE (grades 1 to 4). One patient had grade 4 hypokalemia secondary to GI toxicity. The most common grade 3 toxicities were diarrhea (22%), electrolyte imbalance (15%), thrombocytopenia (15%), dehydration (11%), and nausea (11%). Diarrhea was the most common AE, with all grades occurring in 23 patients (93%): grade 1 in 19%, grade 2 in 52%, and grade 3 in 22% (Table 2). At the RP2D of neratinib (160 mg/d) with full-dose T-DM1, there were no DLTs (zero of eight patients). For other doses of neratinib, there were seven DLTs: one of six patients at 120 mg/d; four of eight at 200 mg/d; and two of three at 240 mg/d (Appendix Table A1, online only). All patients had a baseline LVEF 50% or greater (median, 60%) and were monitored every 3 months. No patients had an LVEF decline below 50%.

**TABLE 2.**
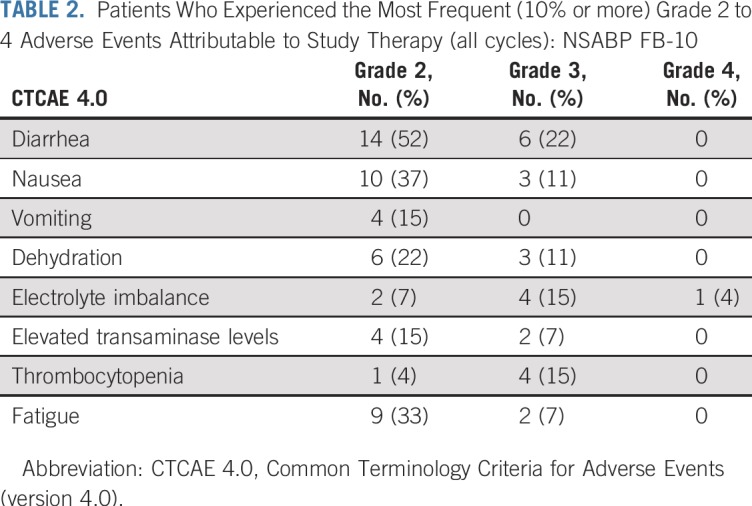
Patients Who Experienced the Most Frequent (10% or more) Grade 2 to 4 Adverse Events Attributable to Study Therapy (all cycles): NSABP FB-10

### Efficacy Assessment

Nineteen patients were evaluable for response. Six were considered nonevaluable because of a DLT in cycle 1 followed by withdrawal (one patient with a DLT continued with a dose reduction and was considered evaluable). One patient was noncompliant, and one had early disease complication before first tumor assessment. Best response was CR in three patients with durations of 364, 510, and more than 969 days; confirmed PR in nine; SD in two; and PD in five.

Of the six patients with known brain metastases, five had PD outside the CNS, and the sixth had PR that lasted 330 days before progressing in the CNS. An additional patient developed new brain metastases at 510 days while in systemic CR. ORs were observed at all doses of neratinib.

### PK

**FIG 1.**
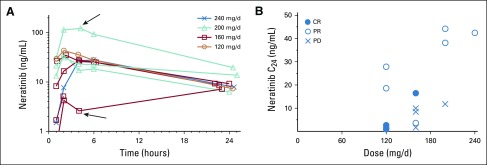
Neratinib pharmacokinetics. (A) Neratinib concentration versus time over 24 hours. The outlier at 200 mg/d (upper arrow) developed a dose-limiting toxicity; the outlier at 160 mg/d (lower arrow) was taking pantoprazole. (B) Neratinib trough concentration at 24 hours (C_24_) on day 1, cycle 2, after dosing, with indication of response. CR, complete response; PD, progressive disease; PR, partial response.

During the 24 hours after the initial dose of neratinib, eight patients had intensive neratinib PK sampling. No dose response was apparent. Two outliers were observed: one, with the highest peak concentration at 200 mg/d, experienced DLT during cycle 1, and the second, with the lowest peak concentration at 160 mg/d, was taking delayed-release pantoprazole (Fig 1A).

Fifteen patients had evaluable steady-state neratinib plasma trough samples collected between 19 and 37 hours after their last neratinib dose. Samples in two patients had values of 0.9 and 1.2 ng/mL, which were below the assay lower limit of quantitation of 2 ng/mL.^13^ Calculation of trough concentrations at 24 hours (C_24_) resulted in median absolute concentration adjustments of 8.3% (range, −20% to 87%). Mean C_24_ was 15.3 ng/mL with 102% coefficient of variation, with large variability of exposure at any given dose (Fig 1B). Dose normalization of C_24_ resulted in a mean of 0.089 ng/mL/mg with 93% coefficient of variation. Patients who experienced PD seemed to have lower trough levels relative to those with responses (*P* = .661 by Mann-Whitney *U* test).^19^ Of note, two of the three CRs also had very low trough exposures.

### Blood Samples for cfDNA

**FIG 2.**
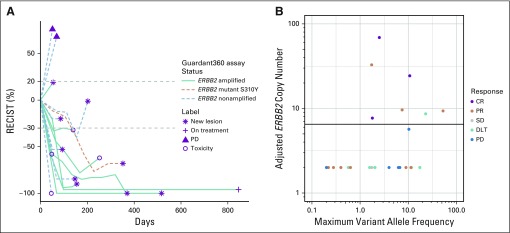
Response Evaluation Criteria in Solid Tumors (RECIST) response and duration. (A) Best response and duration in cell-free DNA in patients with and without *ERBB2* amplification. Although response by dose level is not shown here, objective responses and stable disease (SD) were seen at all dose levels, including two complete responses (CRs) and three partial responses (PRs) in six patients treated with neratinib 120 mg/d, one CR and three PRs in nine patients treated with 160 mg/d, two PRs and one SD in eight patients treated with 200 mg/d, and one PR and one SD in three patients treated with 240 mg/d. (B) Best response above and below the adjusted *ERBB2* copy number threshold of 6.5. DLT, dose-limiting toxicity; PD, progressive disease.

Twenty-seven baseline blood samples were analyzed by Guardant360 assay for *ERBB2* amplification in cfDNA. Nine were *ERBB2* amplified with a copy number cutoff of 2.14 in plasma. One had the *ERBB2*-activating mutation S310Y. Amplification was not detected in the remaining 17 samples. Seven (70%) of 10 patients with amplification or mutation showed CR (n = 3) or PR (n = 4) documented on the second confirmatory imaging evaluation versus four (24%) of 17 without amplification (*P* = .04, Fisher’s exact test).^20^ Furthermore, only PRs and no CRs were observed among those without amplification. Duration of OR in *ERBB2*-amplified patients ranged from 18 to more than 132 weeks; responses in nonamplified patients ranged from 12 to 24 weeks (Fig 2A). Nine blood samples drawn at cycle 2 and two collected at progression were analyzed for *ERBB2* amplification and/or mutations (Table 3). Five of six patients whose plasma samples were initially *ERBB2* amplified had undetectable *ERBB2* amplification by day 1, cycle 2. The sixth sample had an amplification decrease from 6.77 to 2.71 and was no longer amplified at progression. In the patient with an activating *ERBB2* mutation detected on day 1, cycle 1, the mutation was no longer detected on day 1, cycle 2.

**TABLE 3.**
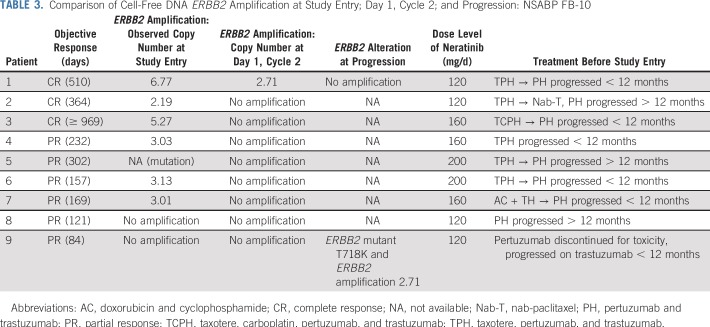
Comparison of Cell-Free DNA *ERBB2* Amplification at Study Entry; Day 1, Cycle 2; and Progression: NSABP FB-10

### Analysis of Tissue Samples for H2T and p95HER2

**FIG 3.**
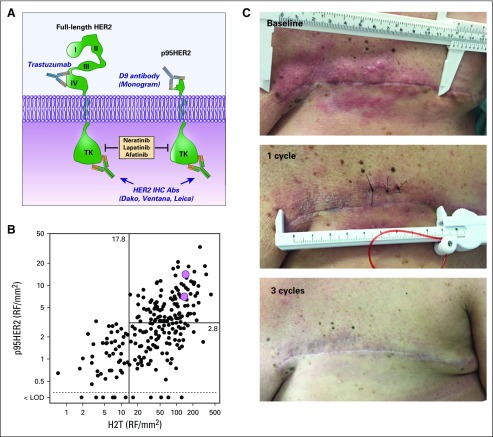
Response in a patient who expressed p95HER2. (A) Anti–human epidermal growth factor receptor 2 (HER2) antibodies from Dako (Agilent, Santa Clara, CA), Ventana Medical Systems (Oro Valley, AZ), and Leica Biosystems (Wetzlar, Germany), currently used for diagnostic immunohistochemical (IHC) evaluation of breast cancer, do not distinguish between expression of full-length HER2 and the truncated p95HER2. In contrast, the antibody developed by Monogram Biosciences specifically detects the truncated p95HER2 form of the receptor and is used in the VeraTag assay. (B) Pink circles indicate the pretreatment total HER2 (H2T) and p95HER2 levels for two patients who achieved complete response (one of whom is represented in the photos in panel C) superimposed for comparison on a previously published scattergram of values obtained from a similar cohort of patients with trastuzumab-treated, metastatic HER2-positive breast cancer (LOD, p95HER2 below the limit of detection). (C) The patient initially presented with locally advanced disease with progressive chest wall disease on trastuzumab plus pertuzumab therapy. By cycle 3, the patient had complete disappearance of chest wall disease and had a clinical complete response. Abs, antibodies; LOD, logarithm of odds; RF, relative fluorescence; TK, tyrosine kinase. Panel B scattergram reproduced with permission.^21^

Two patients with extensive chest wall recurrence had adequate tissue biopsy samples for quantitative analysis of H2T and p95HER2 using the HERmark and VeraTag assays, respectively (Fig 3A), and were well above previously established cutoffs^21^ (Fig 3B). Both patients achieved CR (Fig 3C).

## DISCUSSION

In this study, the DLT of T-DM1 plus neratinib is diarrhea, which is similar to reports of neratinib when used as a single agent or in combination with other agents.^10,22^ Diarrhea is most prominent in cycle 1 and is manageable with primary antidiarrheal prophylaxis. Although no DLTs were experienced at the RP2D of neratinib (160 mg/d), seven of 10 patients experienced early-onset diarrhea, including grade 2 (n = 5) and grade 3 (n = 2) that lasted less than 24 hours. Although the mechanism of neratinib-associated diarrhea is not well understood, preliminary investigations suggested an inflammatory component.^23-27^ One report suggested that budesonide, an oral, poorly absorbed corticosteroid, decreased grade 3 neratinib-associated diarrhea from 50% to 16% and evaluated the addition of the bile salt–binding agent colestipol in mitigating diarrhea.^28^

The median neratinib trough concentration observed was 15 ng/mL (0.09 ng/mL/mg) with a range of 0.9 to 44 ng/mL, which is somewhat lower than that reported previously with 240 mg daily dosing (50 to 60 ng/mL; 0.2 ng/mL/mg).^10^ However, exposure reached preclinical target concentrations of 28 ng/mL, which represents the neratinib half maximal inhibitory concentration for the inhibition of ligand-independent receptor phosphorylation in BT474 cells.^29^ As noted previously, two of three CRs occurred in patients who received the lowest dose of neratinib; these patients also had the lowest trough levels.

The outlying low exposure in the patient who took pantoprazole is consistent with a report of the impact of lansoprazole on maximum serum concentration (reduced by 71%) and area under the curve (reduced by 65%).^30^ Although more frequent DLTs at higher neratinib doses (200 mg and 240 mg) were observed, this was not reflected in peak concentrations of neratinib during the first 24 hours after treatment. Furthermore, responses were seen at all dose levels and steady-state concentrations. In this combination, lower neratinib doses were equally as efficacious as higher, more toxic doses, although any effect of exposure on response might have been obscured by the activity of T-DM1.

An OR to T-DM1 plus neratinib was observed in 12 (63%) of 19 patients, and SD was observed in two patients (one PR was not confirmed on a second imaging study). PD was demonstrated on the first scan in five patients. There are multiple mechanisms of resistance to anti-HER2 therapies in patients treated with previous chemotherapy and dual anti-HER2 interventions. One resistance mechanism seems to be selective elimination of HER2-overexpressing clones, which leaves residual HER2-negative metastatic disease.^31,32^ In patients with HER2-positive metastatic disease treated with trastuzumab-based chemotherapy, 35% showed loss of HER2 amplification in post-therapy tissue biopsy specimens.^31^

Because we have no baseline pretreatment cfDNA determination, we can only speculate that the absence of cfDNA *ERBB2* amplification in 17 (63%) of 27 patients may represent loss of *ERBB2* amplification in patients who received trastuzumab and pertuzumab–based therapy. Although absence of *ERBB2* cfDNA amplification may be partly due to false-negatives, the limited information demonstrated a high degree of concordance between *ERBB2* amplification in cfDNA and in contemporaneous tissue samples.^33^

Our clinical findings suggest that deeper and more durable responses occur in patients in whom *ERBB2* amplification is detected in blood. Six responding patients with cfDNA *ERBB2* amplification had either a reduction in amplification (n = 1) or undetectable amplification (n = 5) by day 1, cycle 2, which suggests that molecular response in cfDNA may provide an early indication of clinical response. These results are consistent with and support the utility of cfDNA molecular response in several studies, including response to neratinib monotherapy in patients with *ERBB2*-mutated but nonamplified MBC.^34^

Three patients achieved a CR. Of note, two patients, who agreed to tissue biopsy, had assays performed for p95HER2 and H2T, which showed that both were highly expressed. This finding suggests that resistance to previous HER2 antibody therapy may have been the result of a high level of expression of the truncated p95HER2, which lacks the extracellular domain essential for the binding of anti-HER2 antibodies. In *ERBB2*-amplified cancers, additional analysis of both p95HER2 and full-length HER2 components may have important therapeutic implications.

T-DM1 has been paired with a number of other agents. For example, in a phase I study of T-DM1 and tucatinib, a reversible HER2-specific TKI, the overall response rate (CR and PR) was 47% (16 of 34 evaluable patients).^35^ Twenty of 57 patients received both pertuzumab and trastuzumab; however, the response in this group of patients was not specified. In another phase I study,^36^ T-DM1 was combined with alpelisib, a phosphatidylinositol 3-kinase α-isoform–specific inhibitor. The overall response rate was 43% (six of 14). Biomarker-driven studies will be needed to determine whether these regimens target specific subpopulations.

In summary, the RP2D of neratinib with full-dose T-DM1 was determined to be 160 mg/d. With appropriate prophylaxis, manageable diarrhea occurred during the first cycle of therapy and abated thereafter. Responses were seen across all dose levels. On the basis of historical data, the combination of T-DM1 plus neratinib seems more active than either agent alone. In addition, our results highlight the potential benefit of cfDNA in personalizing the selection of cancer treatment and in monitoring response as well as genomic evolution under pressure of therapy (eg, eight of 27 patients had a phosphatidylinositol 3-kinase pathway aberration detected in cfDNA). Alterations in the expression of specific HER2 species in *ERBB2*-amplified cancers, including p95HER2, may have therapeutic implications and require further investigation.

## Figures and Tables

**TABLE A1.**
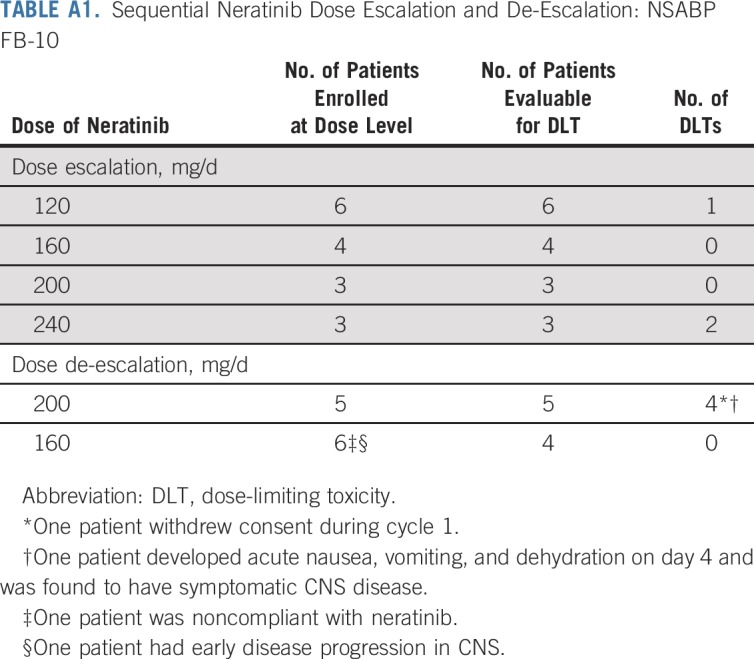
Sequential Neratinib Dose Escalation and De-Escalation: NSABP FB-10
